# The role of preoperative glycemic control in decreasing surgical site infections in lower extremity fractures

**DOI:** 10.1186/s13018-023-04204-7

**Published:** 2023-09-19

**Authors:** Shinsuke Morisaki, Kengo Yoshii, Shinji Tsuchida, Ryo Oda, Tomoya Fuke, Kenji Takahashi

**Affiliations:** 1 Department of Orthopaedics, Saiseikai Shiga Hospital, Ohashi 2-4-1, Ritto, Shiga 520-3046 Japan; 2 Department of Mathematics and Statistics in Medical Sciences, Kyoto Prefectural University of Medicine, Shimogamo Hangi-tyo Sakyo-ku 1-5, Kyoto, 606-0823 Japan; 3 Department of Orthopaedics, Kyoto Prefectural University of Medicine, Kawaramachi Hirokoji, Kamigyo-ku, Kyoto, 602-8566 Japan; 4 Department of Diabetes and Endocrinology, Saiseikai Shiga Hospital, Ohashi 2-4-1, Ritto, Shiga 520-3046 Japan

**Keywords:** Diabetes mellitus, Fracture, Surgical site infection

## Abstract

**Background:**

Postoperative surgical site infections (SSIs) are an important complication to prevent in surgical treatment. Patients with diabetes mellitus (DM) have a higher risk of SSIs. Preoperative glycemic control is required. For patients with orthopedic trauma, the duration of preoperative glycemic control is limited because delaying operative treatment is difficult. However, whether preoperative glycemic control would decrease the risk of SSIs in diabetic patients with lower extremity fractures is unclear. The first aim of this study was to investigate the rate of SSIs among patients with DM who had undergone preoperative glycemic control, compared with that of patients without DM. As the secondary aim, we sought to demonstrate among patients with DM whether preoperative glycemic control would affect the development of SSIs between patients with controlled DM and patients with poorly controlled DM.

**Methods:**

In this retrospective cohort study, 1510 patients treated surgically for lower extremity fractures were enrolled. Data collected were patient age, sex, body mass index, history of DM, development of SSIs, tobacco use, the presence of an open fracture, the period between the day of injury and the operation, the length of surgery, and blood glucose levels on admission and on the day before surgery.

**Results:**

The rate of total SSIs was 6.0% among patients with DM and 4.4% among patients without DM (*p* = 0.31). Multivariate logistic regression revealed a significant association between the development of SSIs and the presence of DM (odds ratio, 1.79; 95% confidence interval 1.01–3.19; *p* = 0.047). The results of the secondary study revealed that the rate of early SSIs was significantly higher in the poorly controlled DM group than in the controlled DM group (5.9% vs. 1.5%; *p* = 0.032). However, multivariate logistic regression revealed that control levels of DM were not significantly associated with the development of SSIs.

**Conclusions:**

Even though patients with DM had undergone preoperative glycemic control, SSIs were significantly associated with DM, especially when the patients had poorly controlled DM. This finding suggested that continuous glycemic control is important preoperatively and postoperatively to prevent SSIs.

## Background

Postoperative surgical site infections (SSIs) are an important complication to prevent in any surgical treatment [1–4]. Reports indicate that patients with diabetes mellitus (DM) have an increased risk of SSIs, compared with patients without DM [5–7]. To decrease the risk of SSIs in patients with DM, preoperative glycemic control is important [8].

Taking sufficient time to attain appropriate glycemic control is possible in elective surgeries such as joint arthroplasty [8–11]. By contrast, for patients with orthopedic trauma, operative treatment is difficult to delay and the duration of preoperative glycemic control is limited [12]. To overcome this obstacle, comanagement with internal medicine and advances in medication enable the successful reduction of blood glucose (BG) levels preoperatively [11, 13].

However, whether preoperative glycemic control would decrease the risk of SSIs when treating lower extremity fractures in patients with DM remains unclear. Furthermore, the risk of SSIs is also influenced by other factors such as a patient’s background, including age, tobacco use, and steroid use, and the presence of an open fracture [2, 4, 14, 15]. The aims of this study were (1) to investigate the rate of SSIs of patients with DM who underwent preoperative glycemic control, compared with that of patients without DM, and (2) to demonstrate whether preoperative glycemic control among patients with DM would affect the development of SSIs between patients with controlled DM and patients with poorly controlled DM.

## Methods

### Study population

This study was a retrospective cohort study. Medical charts were reviewed of 1510 consecutive patients who were managed surgically for lower extremity fractures between June 2016 and May 2021 in one hospital. Clinical data were collected such as age, sex, body mass index (BMI), presence of DM, development of SSIs, tobacco use, presence of open fractures, use of an external fixator, period between the day of injury and the operation, length of surgery, BG levels on admission and on the day before the operation, and hemoglobin A1c (HbA1c) on admission. Patients were excluded who were < 18 years old and had not undergone at least one orthopedic procedure. Patients whose follow-up was less than 1 month were also excluded, including 5 patients with DM and 7 patients without DM. Eight surgeons performed the surgeries.

### Diagnosis of DM

DM was diagnosed by using the patient’s history obtained during the preoperative evaluation. We also included patients with DM if the preoperative screenings met the criteria, which included an HbA1c value ≥ 6.5% or random glucose values ≥ 200 mg/dL [16]. All patients with DM were treated by internal medicine comanagement with nutritional intervention and medication and by using a sliding scale insulin.

For the first analysis, patients with DM were compared with patients without DM (i.e., the control group). For the secondary analysis, we divided patients with DM into two cohorts: the controlled DM group and the poorly controlled DM group, depending on the HbA1c and BG levels on admission. Controlled DM was defined as an admission HbA1c value < 7.5% or random BG levels ≤ 200 mg/dL. Poorly controlled DM was defined as an HbA1c value ≥ 7.5% or random BG levels > 200 mg/dL [16].

### Identification of SSIs

SSIs were identified when a wound required an unplanned intervention such as oral or intravenous antibiotics or an unplanned surgical intervention [1, 10]. SSIs were divided, depending on severity, into superficial SSIs and deep SSIs [16]. Superficial SSIs were identified when additional treatment was necessary such as local wound care or oral antibiotics. Deep SSIs were identified when intravenous antibiotic treatment or surgical debridement was necessary.

We divided the timing of the occurrence of SSIs as “early SSIs” and “late SSIs.” Early SSIs developed within 30 days postoperatively. Late SSIs developed from 30 days to up to 1 year postoperatively.

The rate of SSIs, based on the part of the lower extremity, was also examined. We classified the fractures as “isolated” or “multiple.” Isolated fractures were divided into three groups: pelvis/hip, femur/knee/tibia, and ankle/foot.

### Statistical analysis

The clinical characteristics of the two groups were compared by using Wilcoxon’s rank sum test, the chi-squared test, or Fisher’s exact test. The odds ratio (OR) and 95% confidence interval (CI) were estimated with a logistic regression model. Multivariate logistic regression was performed to analyze dichotomous variables for their associations with the primary outcome (i.e., SSIs) and to analyze any associations between patients with DM and patients without DM. The following variables were examined as potential confounders: age, sex, BMI, tobacco use, open fractures and presence of DM. For the secondary analysis, multivariate logistic regression was conducted among patients with DM only by using the potential confounder of HbA1c on admission instead of the presence of DM. A value of *p* < 0.05 was statistically significant. All statistical analyses were conducted using R version 4.1.2 (R Foundation for Statistical Computing, Vienna, Austria).

## Results

The patients’ characteristics are shown in Table 1. Among the 1510 patients included, 22% (335) patients had DM. Patients with DM were significantly older than patients without DM (81 years vs. 75 years; *p* < 0.001). More women than men were included (931 and 579, respectively; *p* = 0.031). Steroid use was significantly higher among patients with DM than among patients without DM (10% and 5%, respectively; *p* < 0.001). BG levels on admission were significantly higher in patients with DM than in patients without DM (177 mg/dL and 118 mg/dL, respectively; *p* < 0.001). The HbA1c value was higher in patients with DM than in patients without DM (6.4% and 5.5%, respectively; *p* < 0.001). The BG levels in patients with DM on the day before surgery decreased to 135 mg/dL, compared to its levels on admission.

**Table 1:** Comparison of the patients with DM and patients without DM

	With DM	Without DM	*p* value
No. of patients	335	1175	
Age, y	81 (71, 87)	75 (54, 86)	< 0.001
Female sex (vs. male sex)	224 (67%)	707 (60%)	0.031
BMI, kg/m^2^	21.8 (19.3, 24.2)	21.4 (19.2, 23.8)	0.41
Tobacco use	60 (18%)	226 (19%)	0.64
Steroid use	33 (10%)	54 (5%)	< 0.001
Open fractures	18 (5%)	75 (6%)	0.58
Use of external fixator	23 (7%)	106 (9%)	0.26
Period between injury and surgery, days	4 (2, 7)	4 (2, 8)	0.84
Length of surgery, min	81 (59, 114)	79 (58, 110)	0.51
BG levels on admission, mg/dL	177 (137, 225)	118 (105, 136)	< 0.001
HbA1c on admission	6.4 (5.9, 7.2)	5.5 (5.3, 5.7)	< 0.001
BG level on the day before surgery, mg/dL	135 (111, 166)	120 (114, 145)	0.49

Data are shown as the n (%) or as the median (25th percentile, 75th percentile)

*DM* diabetes mellitus, *BMI* body mass index, *BG* blood glucose, *HbA1* hemoglobin A1

The overall rate of SSIs is listed in Table 2. The total rate of SSIs was 6.0% for patients with DM and 4.4% for patients without DM (*p* = 0.31). The rate of early SSIs was 3.3% in patients with DM and 2.0% in patients without DM (*p* = 0.26). The rate of late SSIs was 2.7% in patients with DM and 2.4% in patients without DM (*p* = 0.91).

**Table 2:** Comparison of SSIs between patients with DM and patients without DM

Variable	With DM, *n* (%)	Without DM, *n* (%)	*p* value
SSIs total	20 (6.0%)	52 (4.4%)	0.31
Early	11 (3.3%)	24 (2.0%)	0.26
Late	9 (2.7%)	28 (2.4%)	0.91
Type of SSIs			
Superficial	10 (3.0%)	34 (2.9%)	1.00
Deep	10 (3.0%)	18 (1.5%)	0.13

*SSIs* surgical site infections, *DM* diabetes mellitus

When adjusted for age, sex, BMI, tobacco use, open fractures, and DM, multivariate logistic regression revealed a significant association of SSIs with DM (OR 1.79; 95% CI 1.01–3.19; *p* = 0.047) (Table 3). Furthermore, open fractures were associated with SSIs (OR 5.37; 95% CI 2.94–9.82; *p* < 0.001).

**Table 3:** Multivariate logistic regression analysis for the association of SSIs with DM

Variable	Odds ratio	95% Confidence interval	*p* value
Age, y	0.98	0.97–0.99	0.005
Female sex (vs. male sex)	0.74	0.41–1.33	0.32
BMI, kg/m^2^	1.04	0.98–1.10	0.23
Tobacco use	0.90	0.49–1.65	0.73
Open fractures	5.37	2.94–9.82	< 0.001
DM	1.79	1.01–3.19	0.047

*SSIs* surgical site infections, *DM* diabetes mellitus, *BMI* body mass index

The demographic data of the controlled DM and poorly controlled DM groups are shown in Table 4. Two hundred patients were in the controlled DM group and 135 patients were in the poorly controlled DM group. The percentage of open fractures was significantly greater in the controlled DM group than in the poorly controlled DM group (10% and 2%, respectively; *p* = 0.002). The BG levels were significantly higher in the poorly controlled DM group (237 mg/dL) than in the controlled DM group (146 mg/dL) (*p* < 0.001). The HbA1c value was higher in the poorly controlled DM group (7.2%) than in the controlled DM group (6.1%) (*p* < 0.001). The BG levels in the controlled and poorly controlled DM groups on the day before surgery was decreased to 131 mg/dL and 145 mg/dL, respectively, compared to the levels on admission.

**Table 4:** Comparison of patients with controlled DM and patients with poorly controlled DM

Variable	Controlled DM	Poorly controlled DM	*p* value
No. of patients	200	135	
Age, y	81 (72, 88)	80 (70, 87)	0.28
Female sex (vs. male sex)	131 (66%)	93 (69%)	0.60
BMI, kg/m^2^	22 (19.8, 24.2)	21 (18.8, 24.3)	0.43
Tobacco use	43 (22%)	17 (13%)	0.052
Steroid use	22 (11%)	11 (8%)	0.50
Open fractures	4 (2%)	14 (10%)	0.002
Use of external fixator	15 (8%)	8 (6%)	0.74
Period between injury and surgery, days	4 (2, 8)	4 (2, 7)	0.41
Length of surgery, min	79 (56, 108)	84 (63, 118)	0.091
BG levels on admission, mg/dL	146 (125, 171)	237 (208, 287)	< 0.001
HbA1c on admission	6.1 (5.8, 6.6)	7.2 (6.4, 8.5)	< 0.001
BG levels on the day before surgery, mg/dL	131 (108, 154)	145 (119, 186)	0.013

Data are shown as the n (%) or as the median (25th percentile, 75th percentile)

*DM* diabetes mellitus, *BMI* body mass index, *BG* blood glucose, *HbA1c* hemoglobin A1c

The rates of SSIs in the controlled DM and poorly controlled DM groups are presented in Table 5. The total rate of SSIs was 4.5% for patients with DM and 8.1% for patients without DM (*p* = 0.25). The rate of early SSIs was significantly higher in the poorly controlled DM group (5.9%) than in the controlled DM group (1.5%) (*p* = 0.032). The rate of superficial SSIs and deep SSIs was 1.5% and 3.0% in the controlled DM group. By contrast, the rate of superficial SSIs and deep SSIs was 5.2% and 3.0% in the poorly controlled DM group.

**Table 5:** Comparison of SSIs between patients with controlled DM and patients with poorly controlled DM

Variable	Controlled DM	Poorly controlled DM	*p* value
SSIs total	9 (4.5%)	11 (8.1%)	0.25
Early	3 (1.5%)	8 (5.9%)	0.032
Late	6 (3.0%)	3 (2.2%)	0.74
Type of SSIs			
Superficial	3 (1.5%)	7 (5.2%)	0.10
Deep	6 (3.0%)	4 (3.0%)	1.00

*SSIs* surgical site infections, *DM* diabetes mellitus

To examine the association of the degree of control of DM with the development of SSIs among patients with DM, multivariate logistic regression was conducted. The results revealed that HbA1c on admission was not associated with the rate of SSIs (OR 1.14; 95% CI 0.79–1.63; *p* = 0.48) (Table 6). By contrast, open fractures were associated with an increased rate of SSIs (OR 8.58; 95% CI 1.79–41.1; *p* = 0.007).

**Table 6:** Multivariate logistic regression analysis for the association of SSIs with the HbA1c on admission among patients with DM only

Variable	Odds ratio	95% Confidence interval	*p* value
Age, y	0.96	0.92–1.00	0.066
Female sex (vs. male sex)	0.67	0.20–2.30	0.53
BMI, kg/m^2^	1.04	0.91–1.19	0.57
Tobacco use	1.31	0.36–4.82	0.68
Open fractures	8.58	1.79–41.1	0.007
HbA1c on admission	1.14	0.79–1.63	0.48

*DM* diabetes mellitus, *SSIs* surgical site infections, *BG* blood glucose, *BMI* body mass index

The rate of SSIs, based on the type of fracture, are shown in Table 7. The results were 1369 isolated fractures and 141 multiple fractures. Among the isolated fractures, 858 fractures occurred at the pelvis/hip, 231 fractures occurred at the femoral/knee/tibia, and 280 fractures occurred at the ankle/foot. The SSIs rate was 4.7%, 5.0%, and 3.2%, respectively. No significant difference existed between the groups (*p* = 0.25).

**Table 7:** The difference in SSIs, based on the fracture parts

Injury type	No. of patients	SSI, *n* (%)
Isolated fracture	1369	64 (4.7%)
Pelvic/hip	858	43 (5.0%)
Femoral/knee/tibia	231	13 (5.6%)
Ankle/foot	280	8 (3.2%)
Multiple fractures	141	8 (5.7%)

*SSIs* surgical site infections

## Discussion

We investigated the association of the development of SSIs in lower extremity fractures with the presence of DM among patients who underwent optimization of preoperative glycemic control. In this study, we found that the SSIs were significantly associated with the presence of DM, even when patients with DM had undergone optimization of preoperative glycemic control. Furthermore, no significant association existed between SSIs and the level of control of DM, although the rate of early SSIs in the poorly controlled DM group was significantly higher than that in the controlled DM group. Therefore, continuous glycemic control is important preoperatively and postoperatively to prevent SSIs.

The reason that a significant association remained between DM and SSIs, although the patients underwent optimization of preoperative glycemic control, may be related to the duration between the injury and the operation, which was 4 days, on average. This duration may be too short to improve neutrophil activity and increase inflammatory responses and wound healing [17–19]. However, treating lower extremity fractures is difficult to delay. Therefore, clinicians should understand that monitoring for SSIs should be continued postoperatively, although the importance of glycemic control remains on improving other factors such as malunion, nonunion, and long-term admission [7, 20–22].

Previous literature reports [23–28] have also demonstrated an association of DM with SSIs, which was consistent with our results. The authors report that, in foot and ankle surgery, the rate of SSIs rate was 13.2% among patients with DM and 2.8% among patients without DM [28]. Our average rate of SSIs was comparatively lower than that of previous reports. Our population included individuals with fractures in all parts of the lower extremities and consisted mostly of a single nationality. Other authors also argue that a difference exists between controlled DM and uncontrolled DM [16]. They reported a trend toward a higher rate of SSIs among patients with uncontrolled DM than among patients with controlled DM, which was consistent with our findings.

Multivariate analysis showed that the factor of the presence of open fractures has a significant association with SSIs. Previous studies [29–31] have also shown that an open fracture is a risk factor of SSIs. Therefore, when patients with poorly controlled DM have open fractures, special attention for SSIs should be continued.

This retrospective study has several limitations. It was based on a medical chart review, which contains inherent bias. Different surgeons were involved in the treatment. We classified controlled DM and poorly controlled DM, based on laboratory examinations, without evaluating complications such as neuropathy, retinopathy, and renal disease. However, we examined many risk factors (e.g., age, sex, BMI, tobacco use) that contribute to SSIs, and we attempted to address them by using the proper statistical methods.

## Conclusions

We have proven that SSIs were significantly associated with the presence of DM, even when patients with DM had undergone optimization of preoperative glycemic control. For patients with traumatic injuries, the duration for preoperative glycemic control may be inefficient to improve the immune response. Therefore, surgeons treating lower extremity fractures should maintain continuous glycemic control preoperatively and postoperatively to prevent SSIs.

## Data Availability

The datasets analyzed during the current study are available from the corresponding author on reasonable request.

## Abbreviations

BG: Blood glucose
BMI: Body mass index
CI: Confidence interval
DM: Diabetes mellitus
OR: Odds ratio
SSIs: Surgical site infection
